# Supplementary figures and images for: Drosophila Embryogenesis Scales Uniformly across Temperature in Developmentally Diverse Species
Source: PLoS Genet. 2014 Apr 24;10(4):e1004293. doi: 10.1371/journal.pgen.1004293 (PMC3998915; doi:10.1371/journal.pgen.1004293)

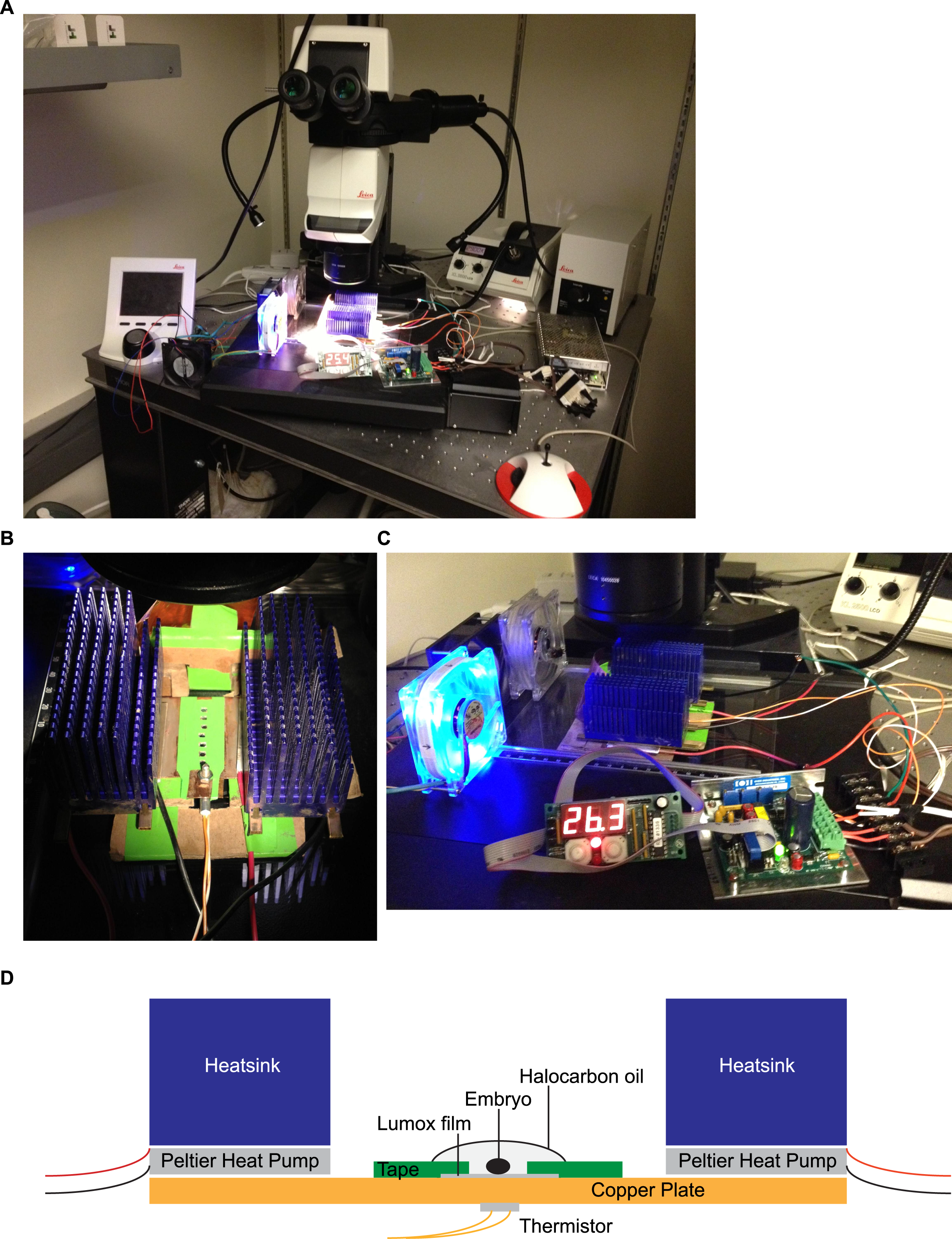

Supplement: Figure S1 — Microscopy imaging setup. (A) The imaging setup, showing the dissecting microscope with temperature control apparatus on the automated stage. (B) A close-up view of the temperature controlled platform flanked by heat-sinks (blue) that sit atop the Peltier thermoelectric controllers. In the center is a copper plate, with a thermister at the bottom to monitor plate temperature. The holes in the green masking tape line up with holes drilled through the copper plate and lined with a gas-permeable membrane. The masking tape helps retain the halocarbon oil. (C) A closer view of the setup. (D) A schematic of the setup demonstrates the temperature control and imaging apparatus in cross-section. (TIF) [file pgen.1004293.s001.tif]

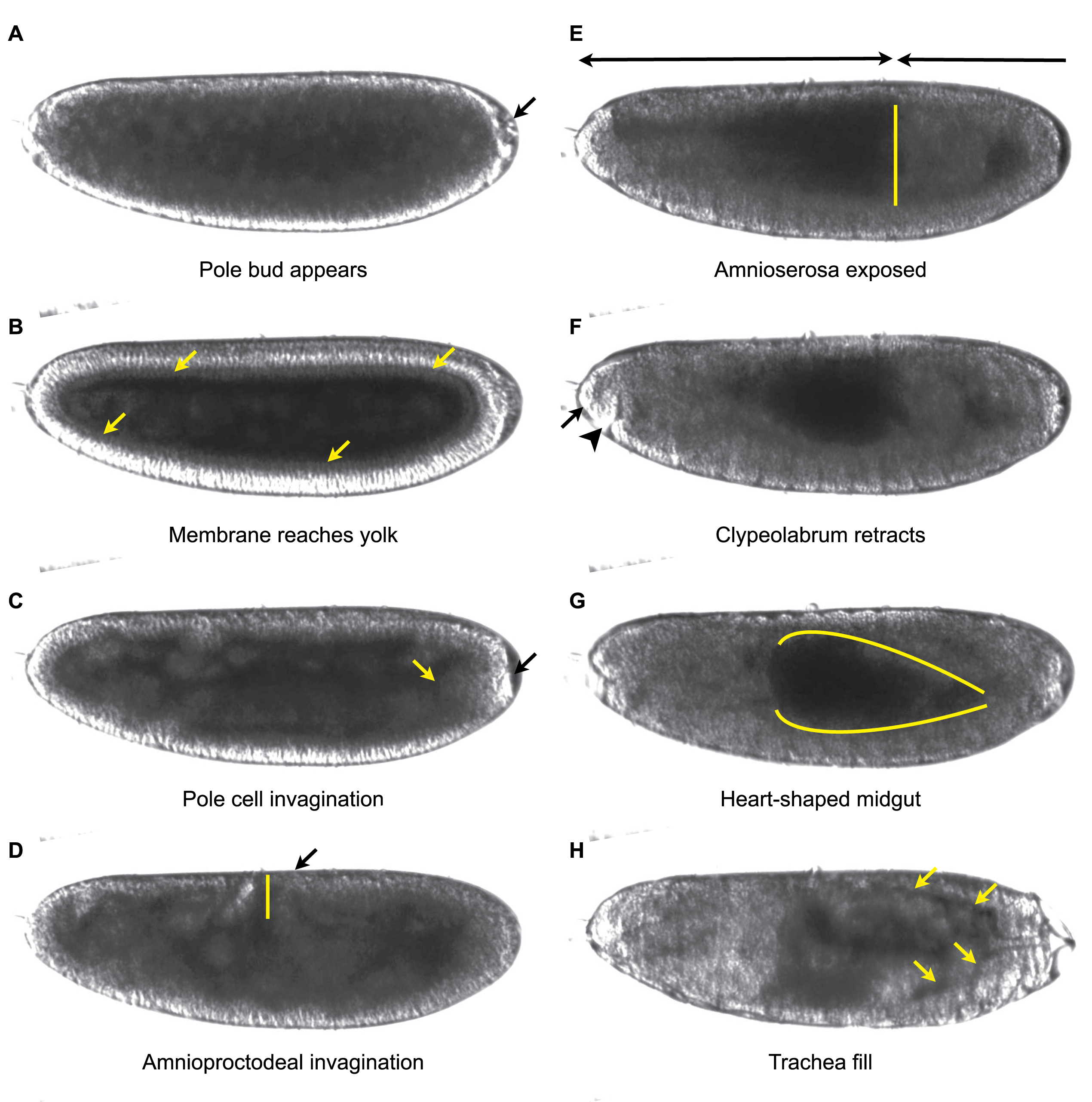

Supplement: Figure S3 — Identifying morphological stages. (A) ‘Pole bud appears’ stage is identified by the first appearance of cells migrating into the posterior gap of the embryo (black arrow). (B) ‘Membrane reaches yolk’ stage is identified by the converging of the leading edge of the invaginating cytoplasmic membrane on the dark yolk. (C) ‘Pole cell invagination’ is identified by the completion of the fold (black arrow) that encapsulates the pole cells (yellow arow). (D) ‘Amnioproctodeal invagination’ is identified by the point when the leading edge of the posterior invagination (black arrow) has covered 80% of the distance to the leading edge of the cephalic furrow (vertical yellow line) and the pole cells have reached the interior of the embryo. (E) ‘Amnioserosa exposed’ is identified by the point when the trailing edge of the germ band has retracted to the posterior 30% of the embryo. (F) ‘Clypeolabrum retracts’ is identified by the withdrawal of the ventral edge of the clypeolabrum (black arrow) from the gnathal buds and vitelline membrane to create a gap (black arrowhead). (G) ‘Heart-shaped midgut’ is identified by the posterior elongation of the formerly spherical developing midgut and residual yolk (dark mass in the center of the embryo) to form a contiguous dark teardrop or heart-shaped mass (delimited with yellow lines). (H) ‘Trachea fill’ is identified by the rapid darkening of the trachea as they fill. The primary branches of the trachea run along the both the left and right dorsal sides, originating at the posterior of the embryo. (TIF) [file pgen.1004293.s003.tif]

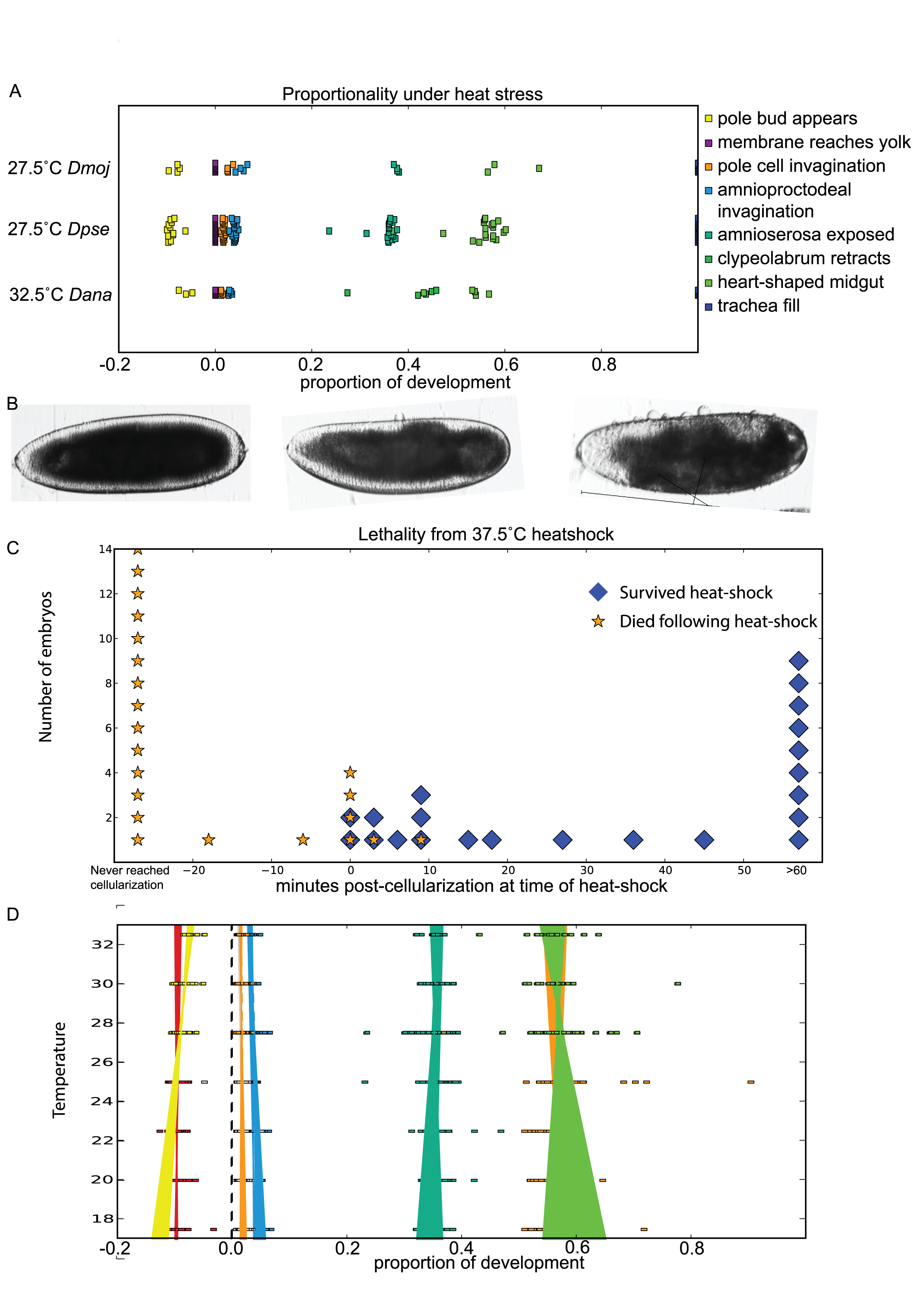

Supplement: Figure S7 — Heat-stress affects syncytial developmental proportionality and morphology. (A) At heat-stress temperatures, the proportionality of developmental stages is affected in some, but not all, embryos. (B) Heat stress in D. melanogaster at 32.5°C affects morphology during yolk contraction and gastrulation. Embryos may exhibit asynchronous yolk-contraction (first image), uneven nuclear distribution during cellularization (second image), or disrupted morphology during gastrulation (third image). (C) Heat shock at 37.5°C for 30 minutes reveals embryos sensitivity prior to the completion of cellularization. Most animals that had completed cellularization survived heat-shock and continued to develop properly (blue diamonds), while no animals that had not completed cellularization prior to heat-shock survived. All embryos that died (orange stars) exhibited severe morphological disruptions. (D) Linear regression of stages across different temperatures reveals that, despite significant variance in later stages (shown in colored bars), only the pre-cellularization time point is affected by heat-stress enough to exhibit a significantly different slope between higher temperatures (27.5°C and above, yellow bar) and lower temperatures (25°C and below, red bar). (TIF) [file pgen.1004293.s007.tif]
